# Cost and time of hospitalization for elderly people with bone fractures in a reference hospital

**DOI:** 10.31744/einstein_journal/2024GS0493

**Published:** 2024-07-10

**Authors:** Aline Cremasco Rocha, Bruna Granig Valente, Danilo Wingeter Ramalho, Juliana Baleki Borri, Carlos Augusto de Mattos, Cintia Kelly Bittar

**Affiliations:** 1 Pontifícia Universidade Católica de Campinas Campinas SP Brazil Pontifícia Universidade Católica de Campinas, Campinas, SP, Brazil.; 2 Hospital PUC-Campinas Celso Pierro Campinas SP Brazil Hospital PUC-Campinas Celso Pierro, Campinas, SP, Brazil.

**Keywords:** Hip fractures, Health care costs, Aged, Pandemics, Length of stay, Costs and cost analysis, Femoral neck fractures, Fractures, bone, Hospitalization

## Abstract

**Objective:**

To describe and analyze the aspects regarding the cost and length of stay for elderly patients with bone fractures in a tertiary reference hospital.

**Methods:**

A cross-sectional retrospective study using data obtained from medical records between January and December 2020. For statistical analysis, exploratory analyses, Shapiro-Wilk test, χ^2^ test, and Spearman correlation were used.

**Results:**

During the study period, 156 elderly patients (62.2% women) with bone fractures were treated. The main trauma mechanism was a fall from a standing height (76.9%). The most common type of fracture in this sample was a transtrochanteric fracture of the femur, accounting for 40.4% of cases. The mean length of stay was 5.25 days. The total cost varied between R$2,006.53 and R$106,912.74 (average of R$15,695.76) (updated values). The mean daily cost of hospitalization was R$4,478.64. A positive correlation was found between the length of stay and total cost. No significant difference in cost was observed between the two main types of treated fractures.

**Conclusion:**

Fractures in the elderly are frequent, resulting in significant costs. The longer the hospital stay for treatment, the higher the total cost. No correlation was found between total cost and number of comorbidities, number of medications used, and the comparison between the treatment of transtrochanteric and femoral neck fractures.

## In Brief

This one-year retrospective study analyzed data from 156 elderly patients with bone fractures undergoing orthopedic surgery at a reference hospital. The authors observed that the longer the hospital stay for treatment, the higher the cost of care. Furthermore, the cost and the number of comorbidities and medications used were not correlated.

## Highlights

**■** Bone fractures were more frequent in women.

**■** The mortality rate among patients was 9.6%.

**■** The main trauma mechanism was falling from standing height.

**■** The total hospitalization cost ranged from R$2,006.53 to R$106,912.74. The mean daily cost was R$4,478.64.

**■** The longer the hospital stay for treatment, the higher the total cost.

## Graphical abstract

**Figure:**
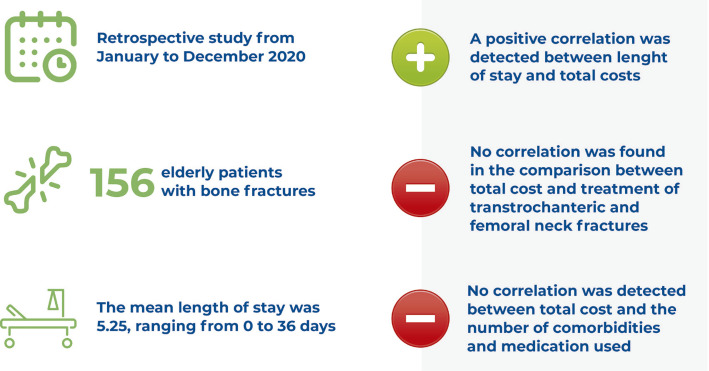

## INTRODUCTION

Fractures in the elderly are associated with high mortality, high frequency, and significant economic cost, especially when their independence is impaired.^(1)^ In the case of the elderly, falls from standing height are the main cause of fractures, whose causal factors can be effectively prevented with specific, well-defined, evidence-based interventions.^(1,2)^

The fragility of the elderly in the face of trauma is due to a decrease in sensory-perceptual capacity, lower speed and reflexes, decreased strength, and decreased bone mineral density.^(3,4)^ It has been observed that this set of factors represents an increased risk of situations unfavorable to health, including dependence; disabilities; a predisposition to slow recovery from acute illnesses, falls, and fractures; and a consequent increase in hospitalization and institutionalization rates.^(5)^

Environmental intervention measures with an analysis of potential dangers effectively prevent falls, up to 39% in high-risk populations, according to a meta-analysis conducted in the United States. In the same study, the authors observed that one or two home visits for environmental interventions are potentially cost-effective, making it possible to avoid expenses from possible falls.^(2)^

In Brazil, between 2000 and 2020, 1.746.097 Authorizations for Hospital Admission (AIHs) due to falls in the elderly were registered in the Hospital Information System of the Unified Health System (SIH/SUS - *Sistema de Informações Hospitalares do Sistema* Único *de Saúde*), with a total cost of more than 2 billion of reais to the health system, according to Lima, et al., thus reinforcing the relevance of the analysis.^(6)^

In 2020, the year of this research, due to the global spread of the novel coronavirus, the pandemic led to the overcrowding of the health system as well as the economy, due to various measures of isolation and social distancing to reduce the peak incidence, deaths, and resource consumption.^(7)^

This work was conducted in an institution to attend the referral flow for traumatology and other non-COVID pathologies in the city of Campinas, state of São Paulo, from April 2020. The effects of this reorganization on other aspects of the population’s quality of life, such as care for chronic and other acute illnesses, are still being studied.

In this scenario, there is a need for analysis, planning, and policies to meet the emerging demands of this population,^(8)^ especially during pandemics, in which, even with the overcrowding of health services, some demands and pathologies remain relevant, such as fractures among the elderly.

## OBJECTIVE

This study aimed to describe and analyze the aspects involved in the costs and length of stay of elderly patients with bone fractures in a tertiary reference hospital and to draw relationships between the cost of care and composing variables such as length of stay, type of fracture, comorbidities, and medications used.

## METHODS

This was a cross-sectional, retrospective, descriptive study carried out in a tertiary hospital located in Campinas, state of São Paulo, with a mixed system, that meets the demands of the Brazilian Public Health System (SUS - *Sistema Único de Saúde*) and the Supplementary Health System (SSS *- Sistema de Saúde Suplementar*). This study was approved and registered by the *Pontifícia Universidade Católica de Campinas* Research Ethics Committee under CAAE: 13390519.6.0000.5481; #3.426.008.

An analysis of the histories of 156 elderly patients diagnosed with a bone fracture by the Department of Orthopedics and Traumatology undergoing at least one orthopedic surgery from January to December 2020 was carried out, a year characterized by the beginning of the community spread of COVID-19 in Brazil. The study institution was classified as a reference for the care of the flow of traumatology and pathologies other than those due to infections with the coronavirus by the Sectorial Coordination of Health of the Municipality of Campinas from April 2020. It received patients from the entire Metropolitan Region of Campinas, covering more than 3.1 million inhabitants in 20 municipalities.

This analysis included patients over 60 years of age at the time of presentation to the service, with a diagnosis of bone fracture, and undergoing at least one orthopedic surgery. All patients agreed to participate in the research by signing an informed consent form to ensure anonymity according to Resolution 196/96 of the National Health Council of Brazil. Patients who did not sign the form, those aged less than 60 years, and those who underwent no surgical procedure were excluded from the analysis.

The variables analyzed were patient age and sex, date of trauma, municipality of residence, mechanism of trauma, comorbidities, medications in use, and type and location of the fracture. In addition, data were collected on the date of orthopedic surgery, type of surgery, total cost of patient care (for paying source, SUS, private, or medical insurance), cost per diem, costs of procedures, number of days of hospitalization, and condition of exit (death or non-death).

Cost data were collected from administrative files and hospital accounts and were composed of auxiliary, diagnostic, and therapeutic services, hospital fees, materials and medications, ICU, per diem, orthoses and prostheses, special materials, anesthesiology, and blood products. Medical fees and indirect costs such as administrative services were not included in the cost analysis. Nominal values, after analyses, were corrected according to corrections of the Extended Consumer Price Index (IPCA - *Índice Nacional de Preços ao Consumidor Amplo*: https://www3.bcb.gov.br/CALCIDADAO/publico/corrigirPorIndice.do?method=corrigirPorIndice) for November 2022.

Exploratory data analysis was performed using descriptive measures (mean, standard deviation, minimum, median, maximum, frequency, and percentage) and graphs were constructed. The Shapiro-Wilk test was applied to check for the normality of the numeric variables, none of which showed a normal distribution. The χ^2^ test was adopted to compare proportions between categories of qualitative variables and to evaluate the relationship between death and age group and between death and sex. The Mann-Whitney U test was used to compare the number of comorbidities between the death and non-death groups. Spearman’s coefficient was used to assess the correlation between the length of stay and total cost. The significance level considered was 5%. Analyses were performed using the R software version 4.2.0, The R Foundation for Statistical Computing.

## RESULTS

Data were collected from 156 elderly patients of both sexes (62.2% female; the frequency of females was significantly higher [p=0.002]) who were treated for bone fractures in 2020. The mean patient age was 76.5 years, range: 60-100 years). The time between consultation and orthopedic surgery ranged from 0 to 15.5 days, with a mean of 2.29 days and a median of 3 days; 25% of patients had their first orthopedic surgery within 24 hours after the first consultation, and 75% of patients had their first surgery within 72 hours after the first consultation.

Among the 156 patients evaluated, 15 (9.6%) died, and the frequency of death was significantly lower than that in non-death (p<0.001) (Table 1). The age group greater than 90 years was more likely to be associated with mortality (p=0.045). Furthermore, no association was found between mortality and sex (p=0.923).

**Table 1:** Association of death with age group, gender, and number of comorbidities

Variable	n	Non-death	Death	p value
Age group, n (%)				0.045*
≤70 years	46	42 (29.8)	4 (26.7)	
71-80 years	52	49 (34.8)	3 (20.0)	
81-90 years	47	43 (30.5)	4 (26.7)	
>90 years	11	7 (4.96)	4 (26.7)	
Gender, n (%)				0.923*
Female	97	87 (61.7)	10 (66.7)	
Male	59	54 (38.3)	5 (33.3)	
Number of comorbidities		1.93 (1.30)	1.70 (1.06)	0.547^†^

* χ^2^ test; ^†^ Mann-Whitney U test.

The mean length of stay was 5.25 days (SD=5.66), ranging from 0 to 36 days, with a median of 3 days. The total cost ranged from R$2,006.53 to R$106,912.74, with a mean value of R$15,695.76 and a median of R$14,032.84. Dividing the total cost by the number of hospitalization days, the mean daily cost of hospitalization was R$4,478.64 (Table 2).

**Table 2:** Measures of position and dispersion of numerical variables

Variable	n	Mean	Standard deviation	Minimum	Median	Maximum
Age (years)	156	76.5	9.8	60.0	76.5	100.0
Time for the first surgery (days)	156	2.29	2.24	0.0	3.0	15.5
Length of stay (days)	156	5.25	5.66	0.0	3.0	36
Total cost (R$)	156	15,695.76	11,924.28	2,006.53	14,032.84	106,912.74
Cost/day (R$)	156	4,478.64	3,033.84	316.64	3,689.40	15,742.67

Patients were grouped into the following age groups: 29.5% between 60 and 70 years, 33.3% between 71 and 80 years, 30.1% between 81 and 90 years, and 7.1% over 90 years. Comparing the frequencies between the age groups, a significant difference was detected (p<0.001); the frequency in the group over 90 years of age was significantly lower than that in the other age groups.

**Figure 1:**
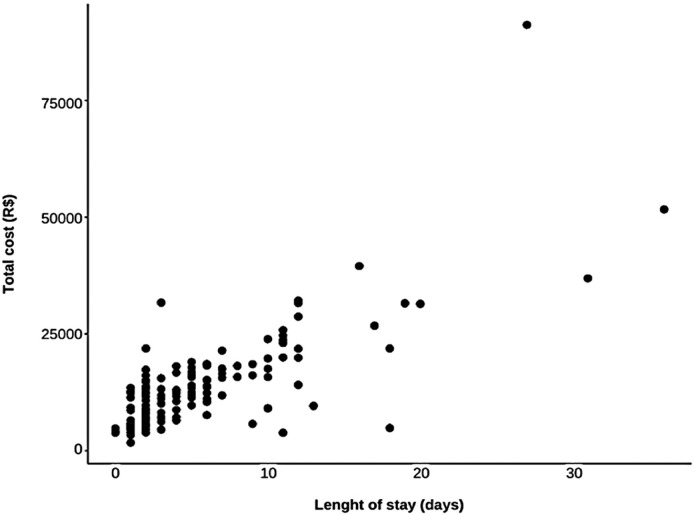
Scatter plot of total length of stay and total cost

Figure 1 illustrates the correlation between the length of stay and total cost. Spearman’s correlation coefficient between the length of stay and total cost was 0.767 (p<0.001), indicating that the correlation between the two variables was positive; that is, the longer the length of stay, the greater the total cost.

It was observed that 14.1% of the consultations came from other cities in the metropolitan region of Campinas, such as Sumaré and Arthur Nogueira (p<0.001).

The main trauma mechanism was falling from a standing height (76.9% of patients), followed by falls from heights (5.1%) and falls from stairs (3.8%). Among the patients, 9.6% had more than one fracture. The femur was the most affected bone (73.7%), followed by the radius and humerus (4.3% each). The most common type of fracture in this sample was a transtrochanteric fracture of the femur (40.4%), followed by a femoral neck fracture (17.3%). In this analysis, for both the site and type of fracture, the two fractures were considered together when the patient had more than one fracture. For analytical purposes, the median cost of hospitalization was calculated only for these two types of fractures, as the others did not present enough observations. The median cost of hospitalization for a transtrochanteric fracture was R$14,499.58 and the median cost of hospitalization for a femoral neck fracture was R$15,526.89. No significant difference was found between the two types of fractures in terms of the total cost of hospitalization (Mann-Whitney U test; p=0.150).

The most common comorbidities were systemic arterial hypertension (SAH) with 38.5%), *diabetes mellitus* (DM) with 11.5%), neoplasms (7.1%), and hypothyroidism (6.4%). In this analysis, no correlation was found between death and the number of comorbidities (p=0.547). In addition, no correlation was observed between the total cost and the number of comorbidities (r=0.166; p=0.116) or between the length of stay and number of comorbidities (r=0.072; p=0.502).

The most commonly administered drugs were losartan (11.5%), simvastatin (11.5%), enalapril (7.1%), AAS (6.4%), and omeprazole (6.4%). No correlation was observed between the total cost and the number of medications used (r=0.196; p=0.107).

## DISCUSSION

Fractures in the elderly, for the most part, originate from low-energy trauma and are triggered by frailty due to the chronic use of medication, reduced physical activity, comorbidities, and age-related diseases such as gait disorders, decreased visual and auditory acuity, and osteoporosis.^(1,3,4)^ Frail individuals are more vulnerable to experiencing events with adverse health outcomes and/or death when exposed to physical stress.^(9)^ In a 10-year retrospective study in Brazil (2008-2018), an overall mean of 224.02 fracture cases per 100,000 elderly individuals was identified.^(10)^ Falls are the main fracture mechanism^(1,4,11)^ and the focus of prevention policies for the elderly population.

In our case, falling from a standing height was the major trauma mechanism, corroborating the medical literature. Some studies show that falls are approximately twice as frequent for women as for men,^(3,6,12)^ data close to those found in this study, in which out of the 156 patients, 97 were women, representing a total of 62.2% of the cases. Estimates indicate that one in three women over the age of 50 years experiences an osteoporotic fracture. Physiological characteristics of the bone and muscle structure, hormonal changes related to menopause and osteoporosis, and the performance of multiple tasks are factors that could explain the greater propensity for falls in females.^(13)^

According to DATASUS, the mean lengths of hospital stay for femoral fractures in elderly people in Brazil were 8.8, 8.5, and 7.5 days in 2018, 2019, and 2020, respectively.^(14)^ In this study, the mean length of hospital stay was 5.25 days, which is within the expected range for the national average. Regarding the time for the first surgery, several studies^(3,15,16)^ have reinforced the importance of early (within 48 hours) intervention, as it is associated with lower mortality, shorter hospital stay, and lower chances of postoperative complications.

In a study carried out in Italy with 71,920 elderly people with proximal femur fractures, early intervention (before 48 hours) was significantly correlated with lower costs and reduced risk of death in the first three months after hospitalization.^(16)^ In a Brazilian study conducted in the metropolitan region of Porto Alegre, Rio Grande do Sul, the longer the time for surgery, the higher the total cost.^(17)^ Our results indicated a similar situation, with a positive correlation between the length of stay and cost. This highlights the potential focus of intervention for strategies that aim to reduce door-to-surgery time.

Losartan and simvastatin were the main medications used by patients. The chronic use of medications and polypharmacy in the elderly is a focus of attention, as the adverse effects of drugs (mainly hypoglycemic, antihypertensive, and psychotropic drugs) can include postural hypotension, drowsiness, dizziness, and increased urination frequency, leading to possible risks for falls.^(18)^

The presence of comorbidities seems to be related to the increased mortality of these patients, in addition to immediate or late postsurgical complications.^(19)^ This data differs from those found in our study, in which there was no correlation between in-hospital mortality and the number of comorbidities. This discrepancy may be because our study did not evaluate long-term mortality. Edelmuth et al. identified hypertension, DM, and heart disease as the main comorbidities in elderly patients with hip fractures.^(19)^

Extrinsic factors include inadequate lighting, slippery and irregular surfaces, unevenness, loose or folded rugs, high or narrow steps, obstacles on the path, and the absence of handrails.^(20)^ Regarding the site most affected by fractures, several national^(4,6,10)^ and international^(3,11,16)^ studies found the femur to be the most affected bone, especially fractures of the proximal third. In our study, femoral fractures (73.7%) were the most common among the 156 elderly people, corroborating the medical literature.

We observed a mortality rate of 9.6% during hospitalization. In a systematic review and meta-analysis of 100 studies from different countries, which analyzed predictors of mortality within 30 days for fractures in the elderly, the median mortality within 30 days was 8% (interquartile range 6.5-9.6%). Heterogeneity was identified among the variables correlated with increased mortality; however, some factors were more correlated with a higher death rate, such as older age, male sex, individuals in ASA strata III-V (American Society of Anesthesiologists scoring), institutionalized patients, and those with comorbidities, such as chronic renal failure, dementia, and DM.^(21)^ In our study, a positive correlation with death was only found in advanced age (>90 years). There was no correlation between death and sex or the number of medications used.

Notably, 14.1% attendances of elderly people with fractures registered by the institution were from other municipalities in the Metropolitan Region of Campinas. These expressive data are due to the reorganization of the municipal health system carried out by the Sectorial Coordination of Access Regulation of the Department of Audit and Regulation of the SUS, in which the PUC-Campinas Hospital became the reference institution for the care of trauma and other non-COVID pathologies in the city of Campinas as of April 2020.^(22)^ This also demonstrates that as Campinas is a reference center in the region in terms of health, it absorbs demand from neighboring cities, overloading the municipal system.^(23)^

During the COVID-19 pandemic, health resources were consumed exponentially, with dynamic resource allocation between institutions, owing to emergency demand.^(24)^ However, fractures in the elderly are a concern that does not significantly interfere with the number of cases, since they are emergencies related to population aging. There were 62,962 hospitalizations in 2019 in Brazil for femur fractures in the elderly, and in 2020, the number was 62,931 (data from DATASUS). The total cost of hospital services for ICD-10 was quite similar in both years, with an increase only in 2021, from 167 million (U$D 31.5 million - using an average dollar to Brazilian real exchange rate of U$D5,29 in November 2022) to 184 million (U$D 34.7 million). The average values per hospitalization remained similar in 2020 and 2021, being R$2,665.64 (U$D 503.52) and R$2,695.27 (U$D 509.11), respectively.^(14)^ In 10 years (2008 to 2018), the total cost was R$1.1 billion for hospitalizations due to femoral fractures in Brazil, an increase of 158% in this period. The cost is projected to increase to R$2.16 billion over the next decade.^(12)^

In this scenario, the costs related to the care of these patients have a great relevance and impact on the hospital’s total costs. The analysis demonstrated that R$2.4 million (U$D 453,334.84) was allocated to direct costs for the treatment of fractures in the elderly in 2020 at the studied institution, with an overall average of R$15,695.76 (U$D 2,964.76). Farias et al. obtained an average cost of R$9,390.21 in 2016 in Paraná, Brazil.^(17)^ In an analysis of proximal femur fractures, Mensor et al. found an average cost of R$5,612.13 per patient for the SUS as the paying source and R$52,384.06 for the Supplementary Health System as the paying source for the period between 2018 and 2021.^(25)^ This cost difference between the paying sources has been noted in several studies. This may explain the high average cost of our service because it is a mixed-source institution (SUS and SSS).

In a retrospective study spanning two decades in the Netherlands (2000-2019), although the length of hospital stay has decreased over the years, care cost continues to increase in the aging Dutch population (as they also involve post-hospital care), despite the reduced incidence of fractures in the elderly.^(26)^

Some factors are related to lower costs for the healthcare system, such as waiting time for surgery.^(16,17,27,28)^ On the other hand, some factors seem to be related to higher total costs, mainly the length of hospital stay. Other characteristics presented heterogeneity among studies, such as the number of comorbidities, medications used, sex, and age.^(27)^ In a Chinese study, higher costs were related to the length of hospital stay, multimorbidities, blood transfusion, and chest injury.^(29)^

The effects of the lack and high price of medical supplies due to the pandemic are still under investigation, since there are few studies (mainly Brazilian) on the subject, and the analyses sought to focus on treatments, efficacy, and safety of medications instead of economic impact.^(30)^ In addition, it is worth mentioning the importance of a complete analysis of costs, adding views on inflation, and readjusting health plans. This justifies the importance of further studies on this topic to analyze the consequences that the economy still suffers from and formalize supplies for strategies and tools for public health.

### Study limitations

The limitations of this study include its retrospective design and single-center design without long-term follow-up. Limitations were also found regarding the discussion of costs for treating bone fractures in the elderly related to the pandemic, owing to the scarcity of studies evaluating the impact.

## CONCLUSION

Fractures occur frequently in the elderly, incurring significant costs, and elderly women are more susceptible to bone fractures. The total cost of treating these patients represents an expressive amount, as this is a pathology related to population aging, it gains higher importance in a pandemic scenario, as the health system is already overloaded due to emergency demand. In the present study, the longer the length of stay, the higher the total cost of care. No correlation was detected between the total cost and the number of comorbidities or medications used. No relationship was found between the total cost of treating transtrochanteric and femoral neck fractures.
